# A Comprehensive Review of Microneedles: Types, Materials, Processes, Characterizations and Applications

**DOI:** 10.3390/polym13162815

**Published:** 2021-08-22

**Authors:** Faisal Khaled Aldawood, Abhay Andar, Salil Desai

**Affiliations:** 1 Industrial Engineering Department, College of Engineering, University of Bisha, Bisha 67714, Saudi Arabia; faldawood@ub.edu.sa; 2 Potomac Photonics, Inc., Halethorpe, MD 21227, USA; aandar@potomac-laser.com; 3 Center for Excellence in Product Design and Advanced Manufacturing, North Carolina A & T State University, Greensboro, NC 27411, USA

**Keywords:** 3D printing, characterization, drug delivery, advanced manufacturing, microneedle, polymers, therapeutics, transdermal

## Abstract

Drug delivery through the skin offers many advantages such as avoidance of hepatic first-pass metabolism, maintenance of steady plasma concentration, safety, and compliance over oral or parenteral pathways. However, the biggest challenge for transdermal delivery is that only a limited number of potent drugs with ideal physicochemical properties can passively diffuse and intercellularly permeate through skin barriers and achieve therapeutic concentration by this route. Significant efforts have been made toward the development of approaches to enhance transdermal permeation of the drugs. Among them, microneedles represent one of the microscale physical enhancement methods that greatly expand the spectrum of drugs for transdermal and intradermal delivery. Microneedles typically measure 0.1–1 mm in length. In this review, microneedle materials, fabrication routes, characterization techniques, and applications for transdermal delivery are discussed. A variety of materials such as silicon, stainless steel, and polymers have been used to fabricate solid, coated, hollow, or dissolvable microneedles. Their implications for transdermal drug delivery have been discussed extensively. However, there remain challenges with sustained delivery, efficacy, cost-effective fabrication, and large-scale manufacturing. This review discusses different modes of characterization and the gaps in manufacturing technologies associated with microneedles. This review also discusses their potential impact on drug delivery, vaccine delivery, disease diagnostic, and cosmetics applications.

## 1. Introduction

### 1.1. Drug Delivery System

Drugs have been delivered in a variety of pathways to improve the quality of health and extend human life. Drug delivery systems have seen drastic improvements from chewing of therapeutic leaves to capsules, pills, injectables, and implantable devices [1]. Over the years, the therapeutic efficacy of a drug has been enhanced by targeting the localized ailment region while reducing its toxic effect to healthy cells [2]. Higher absorption and transport of the drug can be achieved to relieve distressing symptoms for patients.

There are different routes for drug delivery into the human body, which include oral, parenteral, inhalation, transdermal, etc. [3]. The oral route is the oldest route that is convenient for patients with acceptable ease of administration. For long-term medications, the oral route has side effects because it impacts vital organs such as the liver and kidneys. The parenteral route introduces hydrophobic drugs to the human body using intramuscular, subcutaneous, and intravenous pathways [4]. As parenteral drug delivery is a rapid delivery method, it is considered the optimal choice of drug delivery in an emergency [5]. However, the parenteral route can often be quite painful and is thus not a preferred route for many patients. The inhalation route is designed to transport the drug directly to the lungs. This route is painless, comfortable, and designed to target diseases linked with the respiratory systems or certain drugs that are shown to be efficacious via the air–blood barrier [6]. There are however certain disadvantages and risks associated with overdosing through self-administration by the patients that require multiple doses (3 to 4 times) each day [7]. Attempts have been made to further improve dose efficacy and potency of such types of drugs to reduce their risk to patients by identifying optimal material matrices and tunable release kinetics [8,9,10,11,12]. Finally, the transdermal drug delivery (TDD) route focuses on administering drugs through the layers of skin discussed in detail through the next few sections. For example, TDD can be used as an alternative to oral drug delivery in neonates and geriatric patient populations who may often struggle to swallow oral drugs. TDD may also provide a better alternative for protein/peptides/macromolecules to bypass the digestive tract and provide better bioavailability. TDD also has the potential to not directly affect vital organs when delivering potent drugs and provides a mechanism for sustained delivery [13,14,15,16].

### 1.2. Transdermal Drug Delivery (TDD)

**Figure 1:**
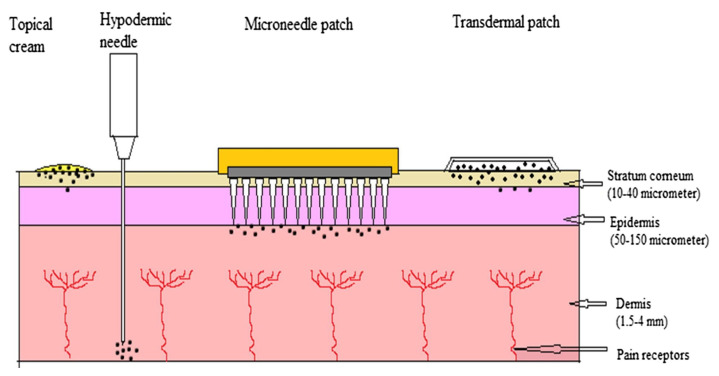
Different types of transdermal drug delivery systems [16]. Reprinted from Biomedicine & Pharmacotherapy, Vol. 109, Tejashree Waghule et al., Microneedles: A smart approach and increasing potential for transdermal drug delivery system, Pages 1249–1258, Copyright (2019), with permission from Elsevier.

Transdermal drug delivery starts with applying the drug directly to the skin. The drug penetrates through the stratum corneum passing through the epidermis and dermis [13]. The drug is available for absorption when it reaches the dermal layer [14]. This method aims to deliver the drug molecules to the bloodstream by controlling diffusion through the skin [15]. Different transdermal drug delivery systems are presented in Figure 1 [16].

**Figure 2:**
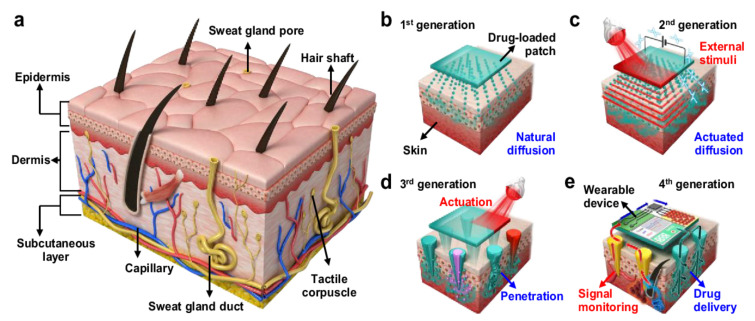
(**a**) Schematic illustration of the human skin. (**b**) First-generation transdermal drug delivery technology via natural diffusion of drugs. (**c**) Second-generation transdermal drug delivery technology for actuated drug delivery via external stimulation. (**d**) Third-generation transdermal drug delivery technology for enhanced drug transport via microneedle-mediated destruction of skin layer and various functionalities accompanying microneedles. (**e**) Fourth-generation transdermal drug delivery technology for patient-customized therapy with the assistance of wearable devices [18]. Reprinted from Advanced drug delivery reviews, Vol. 127, Hyunjae Lee et al., Device-assisted transdermal drug delivery, Pages 35–45, Copyright (2018), with permission from Elsevier.

Prausnitz and Langer have divided the transdermal drug delivery history into four generations as shown in Figure 2 [17]. The first generation focused on providing a low drug load by introducing patch-based technologies using natural diffusion. The second generation focused on using the chemical precursors to actuate drug delivery. The third generation include technologies such as thermal ablation, electroporation, and microneedles, which can precisely target the drug upon entry into the stratum corneum. Finally, the fourth generation involves the combination of sensing modalities along with drug delivery microneedles to control the release of pharmaceutical agents with high precision.

Transdermal drug delivery (TDD) has several advantages over other drug delivery methods. TDD has the ability to deliver the drug to the blood with the desired dosage in a sustained and well-controlled manner [19]. Another advantage of the transdermal route is the reduction of the side effects of drugs by preventing drugs from reaching critical organs such as the liver and kidneys [20]. Moreover, many oral drugs have low bioavailability; this issue can be addressed by using the transdermal drug delivery system [17], specifically for the delivery of macromolecules, peptides, and proteins that typically have low bioavailability via the oral route [21]. Michal Goodman compared transdermal and oral delivery and concluded that the transdermal delivery has a higher safety profile compared to oral preparations in different domains [21].

However, passive transdermal drug delivery is not suitable for drugs with high doses [22] or molecular weights [23], where typically an active penetration enhancer might be needed. Furthermore, transdermal drug delivery may need custom manufacturing and patient-specific formulations which may result in slightly expensive therapy [24]. Perhaps the biggest challenge for TDD is that it has so far been limited to about 22 potent drugs with ideal physicochemical properties and not commercially viable. These include compounds such as nitroglycerine, nicotine, and estradiol, which can passively diffuse and intercellularly permeate through skin barriers to achieve therapeutic concentrations [25]. The stratum corneum is the outermost, biphasic skin layer of 10 to 20 micrometers with both hydrophilic and hydrophobic regions that form the major barrier to limit drug flux into the skin [26]. Significant efforts have been made to enhance transdermal permeation of the drugs across the stratum corneum with the assistance of chemical or physical enhancer [27]. Microneedles provide a physical enhancer or create a physical disruption in the stratum corneum and thus enable the delivery of most drugs through the skin. Once the stratum corneum is breached, drugs can diffuse through the skin once they come in contact with the interstitial fluids. Thus, it is possible to deliver hydrophilic drugs through this method or one may utilize sweat glands as an alternate mechanism. Microneedles address certain key challenges within transdermal delivery such as collapsing veins with a repetitive injection, needle phobia, sustained delivery, etc. Microneedles provide a useful alternative method to avoid these issues [27].

### 1.3. Microneedle (MN) for Transdermal Drug Delivery 

**Figure 3:**
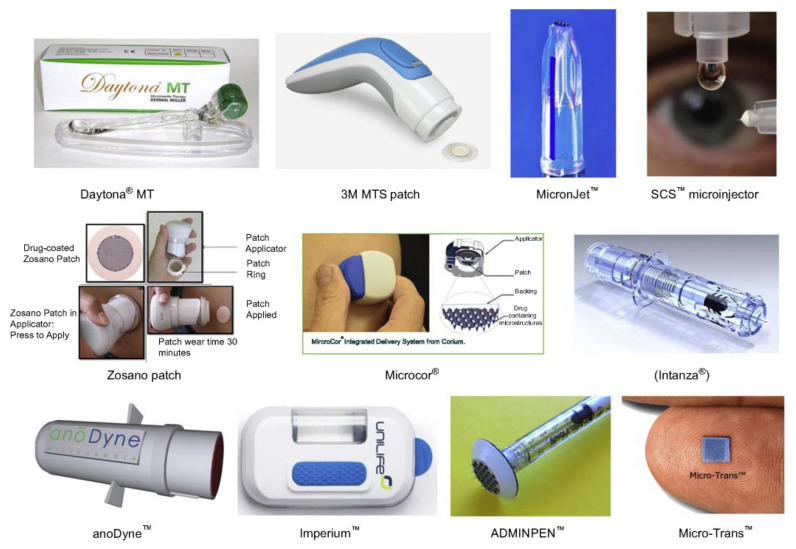
Current microneedle devices (single needle with applicator, microneedles array patch, microneedles pen, microneedle pump patch, and microneedle roller) [36]. Reprinted from Emerging nanotechnologies for diagnostics, drug delivery and medical devices, Rubi Mahato, Microneedles in Drug Delivery, 331–353, Copyright (2017), with permission from Elsevier.

MN technology is a mode of active transdermal drug delivery and is intended to be used a as a replacement to the traditional syringe injections. The MN array is used to penetrate the stratum corneum and deliver the drug with a minimally invasive action [14]. These arrays are micro-sized needles with a height ranging from 25 to 2000 μm [28]. MNs have been used for different applications such as drug and vaccine delivery, cosmetic, and disease diagnostics. MN have various structural arrangements, shapes, forms, and materials along with different manufacturing methods which are further illustrated in this review paper. Figure 3 show some current commercial MN devices. Donnelly et al. argued that 30% of the most recent scientific literature in “transdermal delivery technology” accounted for microneedle studies [29].

The MN drug delivery route can be impacted by external environments such as skin physiology, physiochemical properties, and ambient conditions [30]. These include the relative humidity and temperature in the vicinity of the application area. Too sparse (low humidity) will retard the release of drugs to the skin layers, however too high humidity (such as sweat) can interfere in the drug release kinetics due to excess water and presence of other salts thereby changing the osmotic gradient for transdermal drug delivery. Furthermore, an excess of perspiration can prevent the adhesion of the microneedle patch to the skin further retarding elution of drugs through the skin. Similarly, very low or very high pH ranges around the skin region can result in lower permeability of the drug into the stratus corneum and beyond [31]. Excessive lipid films on the skin form a barrier layer to the stratus corneum and defatting this layer can assist in transdermal absorption [32]. Raising the skin temperature can enhance permeation of drugs due to increase diffusivity and vasodilation of skin vessels [33].

Dosage loading and metering accuracy of microneedles is an important aspect while administering sensitive drugs such as insulin and chemotherapeutics. Typically, microneedle patches require lower dosage as compared to oral ingestion for providing equivalent therapeutic efficacies as digestion and first-pass metabolism are circumvented [18]. The pharmacokinetics of microneedles shows rapid uptake in the bloodstream that can be advantageous for treating localized ailments with much lower drug loading when compared to the oral route. Hollow microneedles serve as drug reservoirs and have the potential to carry higher dosages as compared to solid microneedles. Solid microneedles made from ceramic or metal materials can be coated using inkjet and spray atomization techniques with highly precise drug formulations [34]. The quantity of drug loading for microneedle is highly dependent on the drug type, desired treatment plan, and patient profile. MNs offer a highly precise delivery mechanism due to control of manufacturing processes and drug loading procedures [35]. However, the dissolution of the drug within the skin interfaces can depend on the skin physiology, ambient environmental conditions, and application mode to the skin surface.

#### 1.3.1. Economic Value and Statistics

The economic value of microneedle patches into the current influenza vaccine market in United States was assessed by utilizing a susceptible-exposed-infectious-recovered (SEIR) transmission model [37]. The results show that the incremental cost-effectiveness ratios (ICERs) with healthcare provider administration are less than or equal to $23k per quality-adjusted life years and a market share of 10 to 60% [26]. On the other hand, the ICERs are less than $1.4k for self-administration.

The MN market rose from $5 billion in 2000 to $24 billion in 2013 [38]. By 2025, the market size of global TDD is estimated to expand to about $95 billion [39]. According to a Future Market Insight recent report, by 2030, the MN drug delivery system market will approach $1.2 billion with a Compound Annual Growth Rate (CAGR) of 6.6% [40].

#### 1.3.2. Advantages

A MN is considered to be one of the best ways for transdermal drug delivery due to the fact that drugs administered though this procedure bypass vital human organs such as the liver [41]. Furthermore, it eliminates the pain associated with IV injection by providing a pain-free experience [42]. As a result, it is considered the best choice for people who have needle phobia (trypanophobia). Microneedle transdermal drug delivery application does not require trained personnel thus facilitating ease of use [43]. Furthermore, this reduces the risk of transmitting infection into the body [44].

The stratum corneum acts as a barrier to prevent molecules of any therapeutic agent to pass through the skin and reach the epidermis or dermis layer [16]. A microneedle has the ability to bypass the stratum corneum barrier and deliver the drug into the epidermis or the upper dermis layer without causing any pain [45]. Furthermore, the MN array is long enough to penetrate the stratum corneum and short enough to prevent damage to the dermis or reach nerve endings thus painless [14].

#### 1.3.3. Disadvantages

The use of a microneedle for transdermal drug delivery introduces disadvantages such as extended application time, multiple patches within a given area, requirement of specific mechanical strength, and a good biocompatible material [46]. Rzhevskiy et al. stated that the difficulty in acquiring significant pharmacokinetic data via the MN patch route can impact the dosing parameters and could potentially result in adverse side effects [47]. Bariya et al. argued that MN depth design should also be strongly considered while contemplating the differences in the thickness of the stratum corneum and other layers of the skin from a varied patient populations [48]. The effectiveness of drug delivery and permeation kinetics also depends on the MN device being inserted orthogonal to the skin surface. There is a possibility that the drug dose may escape, or the needles may struggle to penetrate the skin at non-conformal angles. Moreover, repetitive applications of microneedles may result in scarring of the skin surface. There may also be certain drawbacks with respect to the shapes and conformation of needle structures, thus affecting their efficacy. For hollow MNs, for example, their micropores can sometimes get blocked due to compressed tissue for certain skin types, thus affecting their delivery kinetics and penetrability. There are however certain innate drawbacks of using TDD technologies in general that are not specific to just MNs. These include skin irritation, redness, pain, swelling, infection at the application site, etc. [49,50].

## 2. Microneedle History

**Figure 4:**
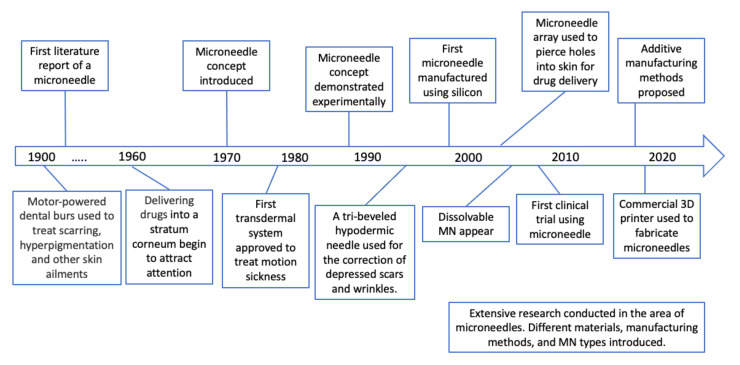
Historic timeline for MN technologies.

Over the years, microneedle concepts have evolved from the use of large needles to the current modern design of microneedles (Figure 4). In 1905, Dr. Ernst Kromayer, a German dermatologist, treated scarring, hyperpigmentation, and other skin ailments by using different sizes of motor-powered dental burs [51]. The first piece of literature that mentions microneedle use was in 1921 by Chambers where he injected the needle into the egg’s nucleus [52]. In the 1960s, delivering drugs by injection into the stratum corneum began to attract attention [53]. Subsequently, the microneedle concept was introduced in the 1970s [54]; however, this concept was not demonstrated experimentally until the 1990s [55]. In 1979, the first transdermal system was approved for use to deliver scopolamine by applying a three day patch to treat motion sickness [17]. In 1994, a subcision surgery was performed by Orentreich where he inserted a tri-beveled hypodermic needle into the skin to release fibrous strands [56]. This surgery targeted the cutaneous defects located under the skin which were responsible for depressed scars and wrinkles. The first microneedle for transdermal delivery was proposed in 1998 and was fabricated from silicon wafers through ion etching and photolithography [57]. The study described the use of microfabricated microneedles for the purpose of enhance drug delivery across the skin. This paper led to extensive research conducted in the microneedle domain. Different materials such as glass, ceramic, metal, and polymers were introduced to fabricate microneedles. In 2004, a microneedle array was used to pierce holes into the skin for transdermal drug delivery [58], which led to several fabrication methods and materials being explored for the purpose of TDD. Solid, coated, hollow, dissolvable, and hydrogel-forming MNs are all different types of MNs. Furthermore, various manufacturing methods such as laser ablation, photolithography, micro-injection molding, etc. These discoveries led to the first reports of a dissolvable microneedle being used for TDD in 2005 [59]. According to clinicalTrials.gov website, to date, 43 clinical trials have been completed using microneedles, with the first microneedle clinical trial completed in 2007 (accessed on 30 June 2021, 5 p.m.). Recently, additive manufacturing methods to fabricate MN molds were developed to provide low cost solutions for micro-mold manufacturing [60,61]. Reports showing the use of commercially available 3D printers to fabricate the MN master mold presented a new age for device fabrication and possibilities for custom built large-volume manufacturing of MNs [62,63].

## 3. Microneedle Types

**Figure 5:**
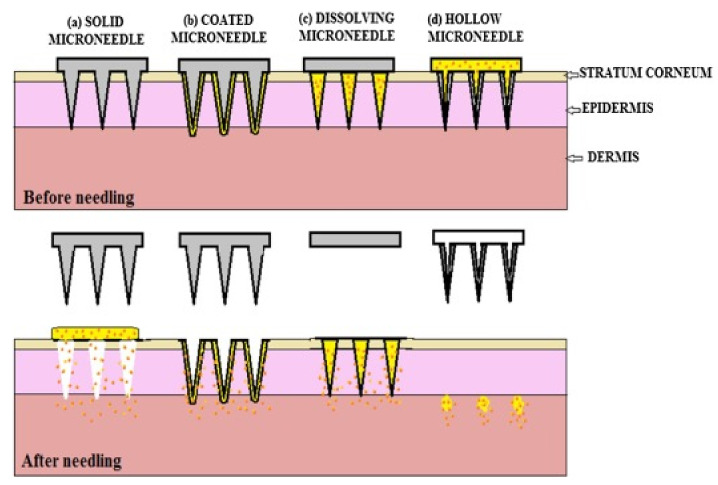
Different types of microneedles: (**a**) Solid microneedles with a poke with patch approach are used for pre-treatment of the skin. (**b**) Coated microneedles use the coat and poke approach, where a coating of the drug solution is applied on the needle surface. (**c**) Dissolving microneedles are made of biodegradable polymers. (**d**) Hollow microneedles are filled with the drug solution and deposit the drug in the dermis [16]. Reprinted from Biomedicine & Pharmacotherapy, Vol. 109, Tejashree Waghule et al., Microneedles: A smart approach and increasing potential for transdermal drug delivery system, Pages 1249–1258, Copyright (2019), with permission from Elsevier.

A variety of materials such as silicon, stainless steel, sugar, and polymers have been used to fabricate solid, coated, hollow, or dissolvable microneedles (Figure 5). Each type of the microneedle has their unique characteristics, advantages, disadvantages, applications, and material type (Table 1).

### 3.1. Solid Microneedle

**Figure 6:**
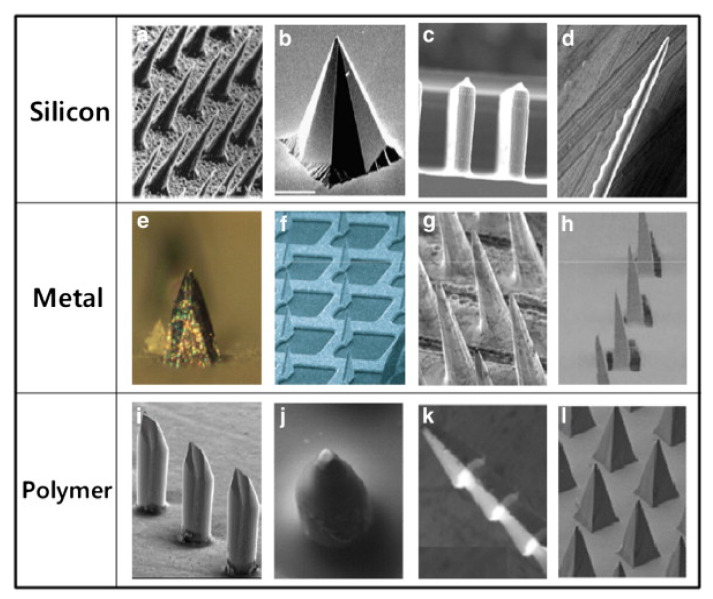
Solid microneedles made of (**a**–**d**) silicon, (**e**–**h**) metals and (**i**–**l**) polymer [41]. Reprinted from Advanced Drug Delivery Reviews, Vol. 64, Yeu-Chun Kim et al., Microneedles for drug and vaccine delivery, Pages 1547–1568, Copyright (2012), with permission from Elsevier.

This type of microneedle structure is designed to penetrate the stratum corneum in order to enhance drug delivery to the dermis to improve the bioavailability and kinetic transport across the skin [64,65]. In comparison to intramuscular delivery, the solid microneedle is suitable for delivery of vaccines as it lasts longer and possesses a more robust antibody response [66]. Solid microneedles are easy to manufacture, have superior mechanical properties, and sharper tips when compared to hollow microneedles [67]. Moreover, the solid microneedle can be fabricated from different materials such as silicon, metals, and polymer (Figure 6) [41].

### 3.2. Hollow Microneedle

**Figure 7:**
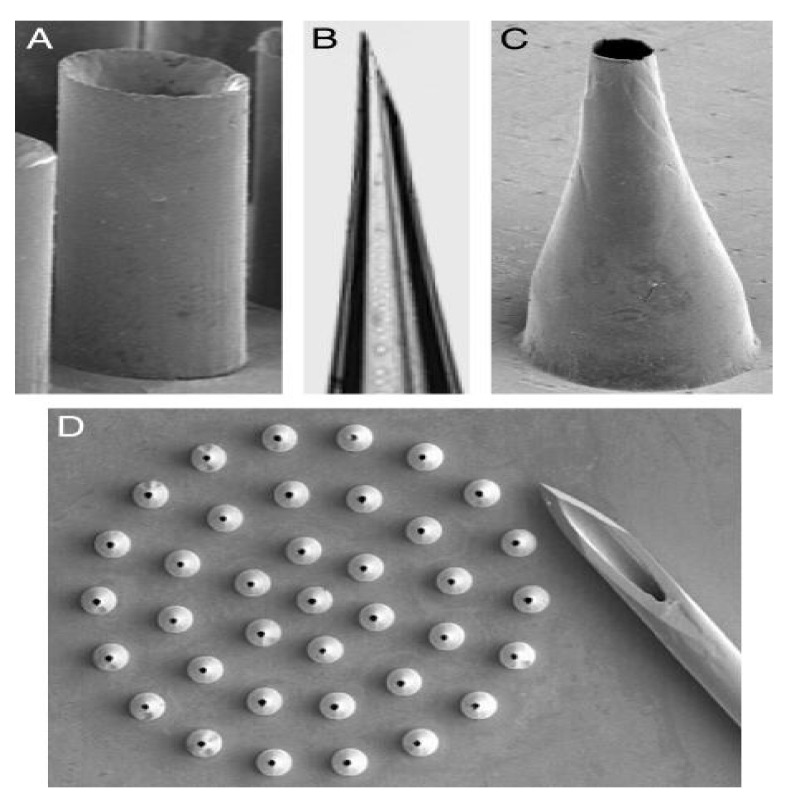
Hollow microneedles fabricated out of silicon, metal, and glass imaged by optical and scanning electron microscopy. (**A**) Straight-walled metal microneedle from a 100-needle array fabricated by electrodeposition onto a polymer mold (200 μm tall). (**B**) Tip of a tapered, beveled, glass microneedle made by conventional micropipette puller (900 μm length shown). (**C**) Tapered, metal microneedle (500 μm tall) from a 37-needle array made by electrodeposition onto a polymeric mold. (**D**) Array of tapered metal microneedles (500 μm height) shown next to the tip of a 26-gauge hypodermic needle [74]. Reproduced with permission from Devin V. McAllister et al., Microfabricated needles for transdermal delivery of macromolecules and nanoparticles: Fabrication methods and transport studies; published by National Academy of Sciences, 2003.

The hollow microneedle consists of a design with a hollow/empty core/chamber in which drug fluid is injected/stored (Figure 7) [16]. Compared to the solid microneedle, the hollow microneedle can handle a large dose/amount of drug solution [68]. A hollow microneedle also has the ability to deliver the drug into the viable epidermis or dermis which is suitable for high molecular weight compounds [69]. Additionally, it controls the drug release over time which makes it suitable for use with liquid vaccine formulations [70]. Unlike solid microneedles, which primarily elute drugs based on the osmotic gradient, hollow microneedles are an active drug delivery system forming a conduit for drug diffusion into the dermis based on a non-pressurized drug reservoir. Both material formulation and fabrication parameters of hollow microneedles can be leveraged to enable tunable release kinetics. Higher concentration drugs can result in burst release drug profiles, whereas matrix-loaded drugs can enable a steady-state drug release lasting days to weeks depending on the application intent [71]. Analogous to hypodermic needles, hollow microneedles can be designed to permit modulation of flow rate and pressure. Process parameters such as microneedle aspect ratio (height to base diameter ratio) can be controlled for rapid release, slow infusion, or time-varying delivery rate [72]. Over the years, the hollow microneedle has successfully been applied to a variety of vaccine/inoculations [59]. However, this type of microneedle received less attention compared to the solid microneedle as the hollow microneedle is relatively weaker and requires intensive care in terms of needle design and insertion method [73]. Furthermore, the hollow microneedle suffers from technical difficulties such as leakage and clogging during the injection process [59].

### 3.3. Coated Microneedle

**Figure 8:**
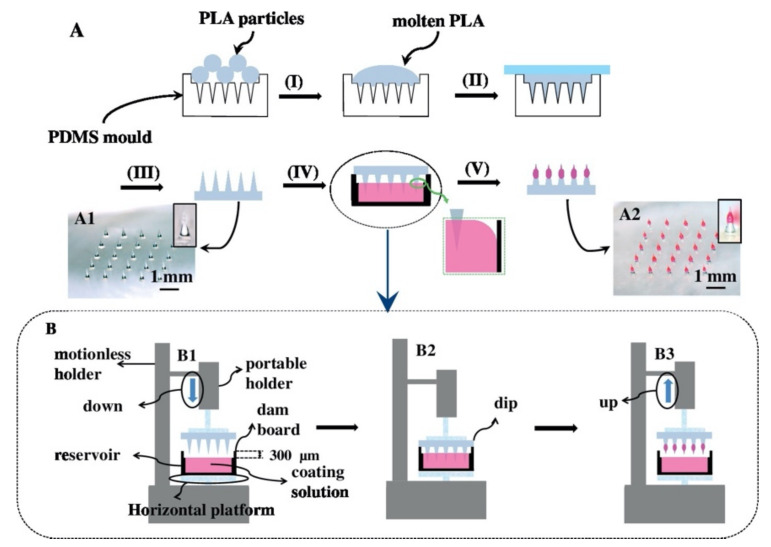
Fabrication of the coated polymer MNs: (**A**) A schematic diagram of the process to fabricate the coated polymer MNs. The coated polymer MNs were fabricated by (I) covering up the surface of the polydimethylsiloxane (PDMS) cavities with heated and melted PLA, (II) filling the mold cavities with melted PLA, (III) exerting pressure on the melted PLA and cooling it down to room temperature, (IV) dipping the coating solution using PLA MNs, and (V) drying the coated polymer MNs. Image (A1) is an image of the 650 μm long PLA MNs. Image (A2) is an image of the 650 μm long MNs coated with formulation III. Image (**B**) is a schematic diagram of the adjustable apparatus that can be lifted and lowered. Image (B1) shows the portable holder with the PLA MNs descending into the reservoir. Image (B2) shows the PLA MNs dipped in the coating solution, and image (B3) shows the portable holder rising from the reservoir [79]. Reprinted from Journal of Controlled Release, Vol. 265, Yang Chen et al., Fabrication of coated polymer MNs for transdermal drug delivery, Pages 14–21, Copyright (2017), with permission from Elsevier.

The coated microneedle is a solid-type MN coated with a drug solution (Figure 8). Typically, it carries a smaller amount of the drug depending on the thickness of the coating layer [75]. The success of delivering drug using a coated MN depends on the ability to reliably coat a controlled drug layer onto MNs [76]. A coated MN has the ability to deliver proteins and DNA in a minimally invasive manner [77]. An advantage of a coated MN is rapid delivery of the drug to the skin; however, the remnant drug at the tip of the needle might infect other patients [78]. Finally, the results of the delivery of the vaccine using coated MN were similar to vaccines using intradermal and intramuscular routes [59].

**Table 1:** Overview: Microneedle Types.

MN Type	Characteristics	Advantages	Disadvantages	Application	Material	References
Solid	Creates channels in the skin to allow drugs reach the lower skin layer. Adequate mechanical strength. Sharper tip.	Allows more drugs to pass into the skin. Easy to manufacture.	Damage to the skin and microincisions need to be closed to avoid infections.	Drug delivery Cosmetic	Silicon Metal Polymer	[41,64,65,66,67]
Hollow	Empty shape to be filled with the drug. Ability to control drug release over time.	Handles a large dose/amount of drug solution.	Weak needles. Requires intensive care in terms of needle design and insertion method. Might cause leakage and clogging.	Disease diagnosis	Silicon	[16,59,68,69,70,71,72,73,74]
Coated	Carries less amount of the drug due to the design. Ability to deliver the proteins and DNA in a minimally invasive manner.	Deliver the drug quickly to the skin.	Prone to infection	Drug delivery Vaccine delivery	Silicon	[59,75,76,77,78]
Dissolving	Facilitates rapid release of macromolecules.	Ease of administration for patients with one step application.	Requires technical expertise to manufacture. Takes time to dissolve.	Drug delivery Cosmetic Vaccine delivery	Polymer	[16,59,69,80,81,82,83]

### 3.4. Dissolving MN

**Figure 9:**
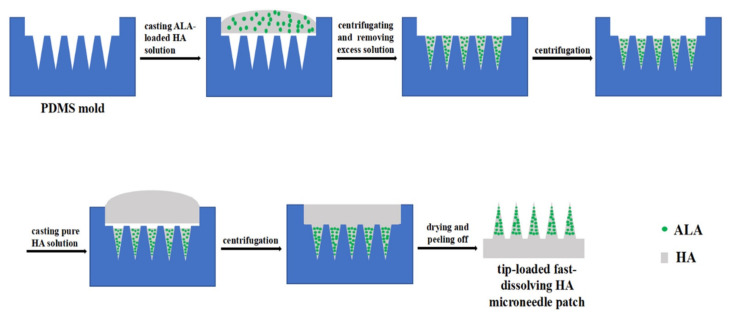
Schematic illustration of the process to fabricate the tip-loaded fast-dissolving HA MN patch [84]. Reprinted from Journal of Controlled Release, Vol. 286, Xiao Zhao et al., Tip-loaded fast-dissolving MN patches for photodynamic therapy of subcutaneous tumor, Pages 201–209, Copyright (2018), with permission from Elsevier.

The dissolvable MN first appeared in 2005 [59] and is a promising technique based on its characteristics. These characteristics include facilitating the rapid release of macromolecules [80], and a one-step drug application which promulgates the ease of drug administration [81]. Due to improvement observed in applying dissolvable MNs following “poke-and-release”, this approach is consider better than other approaches [82]. The dissolvable MN tip can be loaded in a timely manner via a two-step casting method (Figure 9) [81]. Upon insertion of the dissolvable MN into the skin, the drug-load releases and diffuses easily by dissolution of the needle tip [59]. Water-soluble materials are most appropriate for the manufacture of the dissolvable MN [83]. Likewise, the micro-mold method of fabrication is most suitable for the production of the dissolvable MN [69]. The design and production of a dissolvable MN array requires technical expertise [59]. However, this type of MN requires complete insertion which is often difficult to accomplish, and also undergoes a delay in dissolution [16].

## 4. MN Material

**Figure 10:**
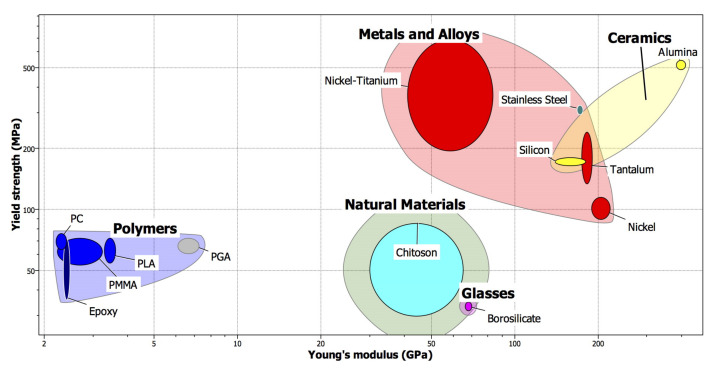
Yield strength vs. Young’s modulus of different materials used for the fabrication of MNs. Plastics: PC, Epoxy, PMMA, PGA, PLA. Metals and alloys: nickel, stainless steel, tantalum, and nickel–titanium. Ceramics: alumina and silicon. Chitison and Borosilicate glass [99]. Reprinted (adapted) with permission from (Cahill, Ellen M., and Eoin D. O’Cearbhaill. “Toward biofunctional MNs for stimulus responsive drug delivery.” Bioconjugate chemistry 26, no. 7 (2015): 1289–1296). Copyright © 2021, American Chemical Society.

The primary reason behind the production of MNs is their ability to penetrate the skin without breaking or bending. Several factors, such as material, manufacturing method, and design, have been considered in tackling the MN manufacturing challenge. A variety of materials have been used to fabricate different types of MNs (Figure 10). Examples of these materials are silicon, metals, ceramic, and polymers [84,85,86,87,88] (Table 2). A combination of different material types have been utilized for biomedical applications in the area of delivery drugs, tissue engineering, and biomedical implants [89,90,91,92,93,94,95,96,97,98].

### 4.1. Silicon

In the 1990s, the first MN was fabricated from silicon material [100]. Silicon possesses numerous advantages over other materials, including its innate flexibility, which allows for easy manufacturability in terms of desirable shapes and sizes of MNs. Silicon has been used to fabricate solid, hollow, and coated MNs [53]. On the other hand, there are limitations associated with using silicon such as time-consuming fabrication [101], high cost [29], and the possibility of causing fractures in the skin [102].

### 4.2. Metal

Metals are utilized in the manufacture of MNs as they have good biocompatibility and mechanical properties [103]. Metals have high fracture toughness [104] and yield strength values. Compared to silicon, metals are stronger and harder to break [16]. The first metal utilized in the fabrication of a MN was stainless steel [105] followed by titanium. Despite the ability of metal MN to pierce the skin, the application of metal MN might cause an allergic reaction [14].

### 4.3. Ceramic

Due to their superior chemical properties and compression resistance, ceramic materials such as alumina have been used to fabricate a MN [106]. However, alumina possesses a lower tensile strength compared to other materials. Calcium sulfate dihydrate and calcium phosphate dihydrate are additional types of ceramics utilized in the fabrication of MNs [16]. A micro-mold technique can be used to fabricate a MN using ceramic material. This technique offers scaled-up production at low cost [107]. A study conducted by Bystrova et al. showed that MNs fabricated from alumina fractured upon manual application to the skin [108].

### 4.4. Polymer

Polymers offer a promising material alternative for MN. They have excellent biocompatibility, low toxicity, and low cost [109]. However, they also possess lower strength compared to silicon and metals [104]. Polymers are usually employed in the production of dissolvable and hydrogel-forming MN’s arrays [71], solid, coated, and hollow MN arrays [53]. Various types of drugs have been applied to the skin using biodegradable MNs [110]. Types of polymers used in the fabrication of MNs included poly (methyl methacrylate) (PMMA), polylactic acid (PLA), poly (carbonate), polystyrene, and SU-8 photoresist [16].

**Table 2:** Overview: MN Materials.

MN Type	Advantages	Disadvantages	Manufacturing Method	MN Type Fit	References
Silicon	Flexible enough to manufacture desirable shapes and sizes.	Time-consuming fabrication. High cost. Possibility of skin fracture	Etching	Solid Hollow Coated	[29,53,55,100,110,111,112]
Metal	Good biocompatibility and mechanical properties. High fracture toughness Strong and hard to break.	High startup cost. Required post-fabrication process. May cause an allergic Reaction.	Laser ablation Etching Injection mold	Solid Hollow	[14,16,101,102,103,113,114,115]
Ceramic	Possesses chemical and compression resistance.	Low tension strength	Micromolding Lithography	Solid Hollow	[16,104,105,116,117]
Polymer	Excellent biocompatibility. Low toxicity. Low cost.	Low strength	Lithography injection molding Casting Laser ablation	Solid Hollow Coated Dissolving	[16,53,71,106,107,108,109,114,118,119]

## 5. MN Manufacturing Method

There exist several methods of fabricating the MN arrays. The most common methods are laser ablation, micro-molding, additive manufacturing, injection molding, chemical isotropic etching, surface/bulk micromachining, and lithography-electroforming-replication (Table 3).

### 5.1. Laser Ablation

**Figure 11:**
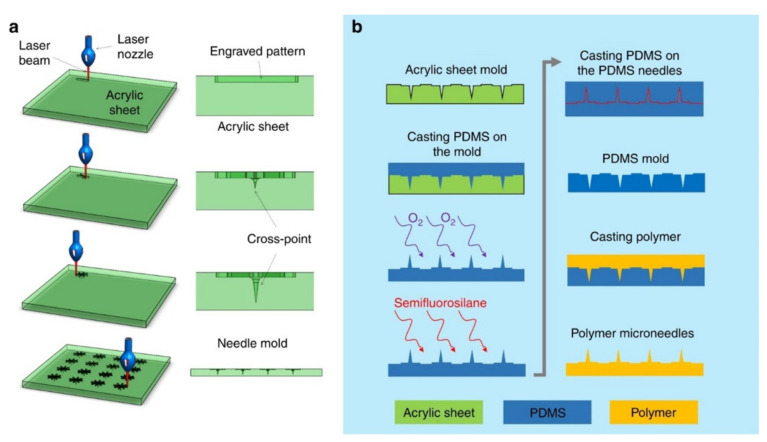
Fabrication of MN mold: (**a**) CO_2_ laser cutter was used to fabricate MN acrylic mold using the proposed cross-over lines (COL) technique. (**b**) The acrylic mold was used to fabricate polydimethylsiloxane (PDMS) MNs mold, which can be used to fabricate a variety of polymer-based MNs [125]. Reproduced with permission from Hojatollah Rezaei Nejad et al., Low-cost and cleanroom-free fabrication of MNs; published by Springer Nature, 2018.

Laser ablation incorporates the use of a focused optical light beam in eliminating material from a substrate to create MN arrays. Lasers have been used to process different materials ranging from micro- and nano-scale for several applications [111,112,113,114,115,116,117,118,119,120,121,122,123]. Various laser types have been studied for the manufacture of MN arrays. These include CO_2_ [124,125] (Figure 11), UV excimer [126,127], and femtosecond laser machine [128]. The laser ablation method is considered an effective and fast method for MNs fabrication. The laser beam takes 10 to 100 nanoseconds to approach the burn point in the material sheet [109]. Laser could also be used to shape any metal. This method is associated with thermal effects at the cutting surface that result in the alteration of MN structure and mechanical properties [129]. This might lead to undesirable effects in MNs such as cracking, or fatigue resistance [130]. The laser ablation method is a non-contact process [131] and subjects low heat loads to the substrate [132]. However, the cost of the laser is higher compared to other types of equipment [109]. The laser ablation method is not suited for large scale manufacturing [129].

### 5.2. Lithography

**Figure 12:**
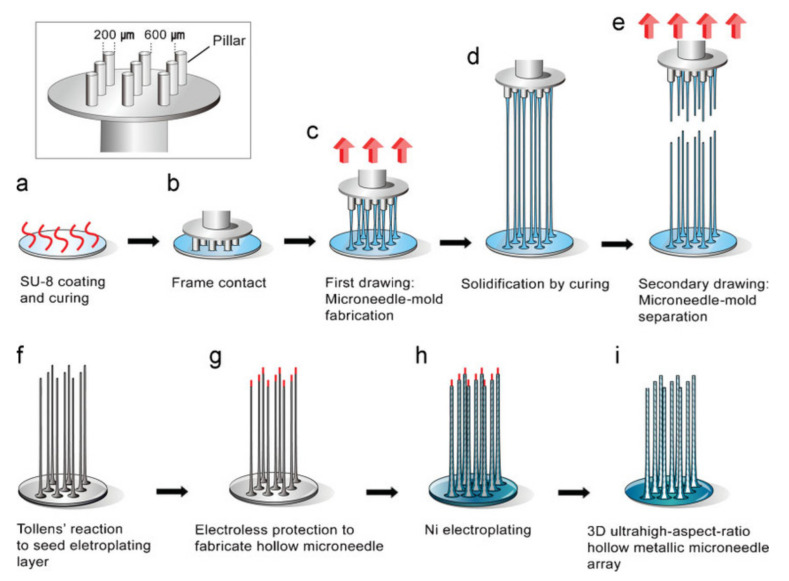
Drawing lithography to produce a 3D UHAR MN. The inset shows a drawing system with patterned pillars for drawing lithography. Stainless drills with a diameter of 200mm and a length of 3 mm were used as pillars and fixed in a 3 × 3 array on a PDMS frame. (**a**) The SU-8 2050 photoresist was spin coated and cooled. (**b**) After the photoresist contacted the patterned pillar, drawing lithography was performed. (**c**) Drawing caused the appearance of an extended conical-shaped bridge between the substrate and pillar. (**d**) The desired UHAR micro-needle mold was cured to generate a rigid structure. (**e**) The separation of the 3D microstructure bridge produced a solid MN mold. (**f**) Chemical deposition on the solid MN molds. (**g**) The upper portion of the MN mold was coated with electroless material using a drawing system. (**h**) Nickel electroplating on conducted solid MN molds. (**i**) The hollow metallic MN array was created upon elimination of the electroless protection and the photoresist MN mold [145]. Reproduced with permission from Kwang Lee et al., Drawing Lithography: Three-Dimensional Fabrication of an Ultrahigh-Aspect-Ratio MN; published by John Wiley and Sons, 2010. Copyright © 2021 WILEY-VCH Verlag GmbH & Co. KGaA, Weinheim.

The lithography technique is used to transfer the master pattern of the geometric shapes onto a surface of a substrate [133,134,135,136,137,138,139,140]. Photolithography is primarily used for pattern transfer due to its wide applicability in the field of microelectronics [141]. Other techniques such as microelectronic and micromachining use lithography as the first step in fabricating a MN [53]. Lithography requires precise processing of the photoresist [142]. This technique contributes to approximately 30–35% of costs for manufacturing integrated circuits [143]. Lithography possesses the ability to create products from a variety of materials such as glass, metal, ceramics, and plastics [144]. It also produces precise geometries and smooth vertical sidewalls [109] (Figure 12). However, this technique requires an advanced facility (cleanroom) and extended production time [125].

### 5.3. Micro-Molding

**Figure 13:**
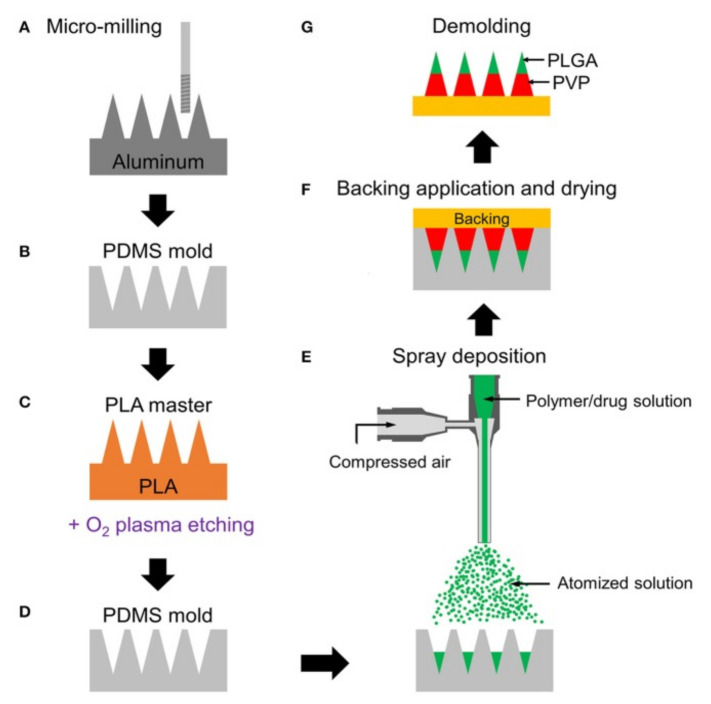
Schematic fabrication process of multilayer MNs: (**A**) Aluminum master fabrication using micro-milling. (**B**) Replication of PDMS mold from the master. (**C**) Fabrication of PLA master by micromolding and tip-sharpening using oxygen plasma. (**D**) Replication of PDMS mold from the PLA master. (**E**) Spray deposition of drug-containing polymer solution to fill the mold cavity. Multilayer MN is formed by sequential deposition of polymer solutions. (**F**) Application of backing material (yellow) on the mold and drying at room temperature for polymer solidification. (**G**) Demolding solidified multilayer MN array from the mold. Green and red represent PLGA and PVP layers, respectively [147]. Reproduced with permission from Min Jung Kim et al., Fabrication of Circular Obelisk-Type Multilayer MNs Using Micro-Milling and Spray Deposition; published by Frontiers in Bioengineering and Biotechnology, 2018.

The micro-molding process consists of making replicates of the master mold. The mold is casted with a solution containing a polymer and active pharmaceutical substances [14] (Figure 13). Micro-molding is considered a cost-effective method and is used for mass production [146]. Micro-molding is commonly used with polymer material for MN fabrication [147]. The PDMS has several advantages in micro-molding techniques such as low cost, ease of use, low surface energy, and thermal stability [148,149]. The limitations associated with this technique are difficulties associated with controlling the depth of penetration, drug load capacity, and mechanical behavior of the polymer [147].

### 5.4. Injection Molding

**Figure 14:**
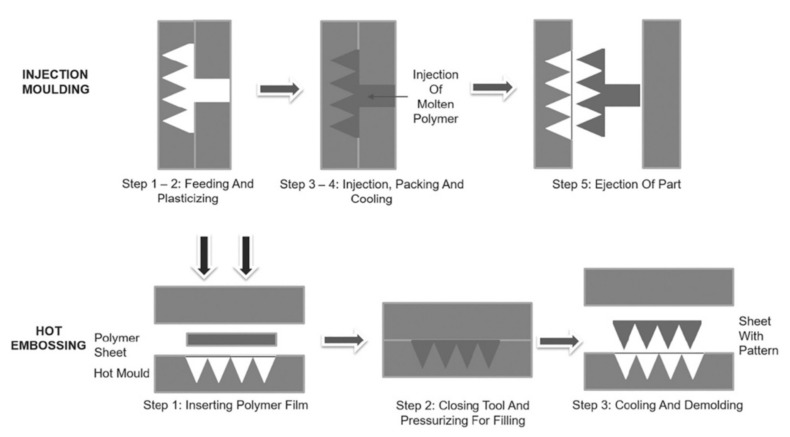
Process steps of standard injection molding and step-and-repeat hot embossing [152]. Reproduced with permission from Herwig Juster et al., A review on microfabrication of thermoplastic polymer-based MN arrays; published by John Wiley and Sons, 2019. copyright © 2021 Society of Plastics Engineers.

Injection molding is another MN fabrication method. The process of fabricating MNs using injection molding and the hot embossing technique is shown in Figure 14. Lhernould et al. used poly carbonate (PC) material to fabricate a 4 × 4 hollow polymer MN array [150]. The MNs were shown to withstand high force and were used for multiple insertions without blunting the needle. Another study used a micro-injection molding process to fabricate a solid MN [67]. These needles could deliver hydrophilic-high molecular weight molecules. Sammoura et al. fabricated a polymeric MN by molding plastic material [151]. The needles were used to successfully penetrate a fresh chicken leg and beef liver and ~0.04 μL of liquid was drawn from these tissues. The proposed method allows the mass production of MNs at low cost.

Micro-injection molding also offers high repeatability, accurate dosing, and high injection flow rates when separating the plasticization and polymer melt injection [80]. The limitation of applying injection molding technique is controlling the small shot size due to the common size of screw which is approximately 15–150 mm and higher initial cost of the equipment [152].

### 5.5. Additive Manufacturing

**Figure 15:**
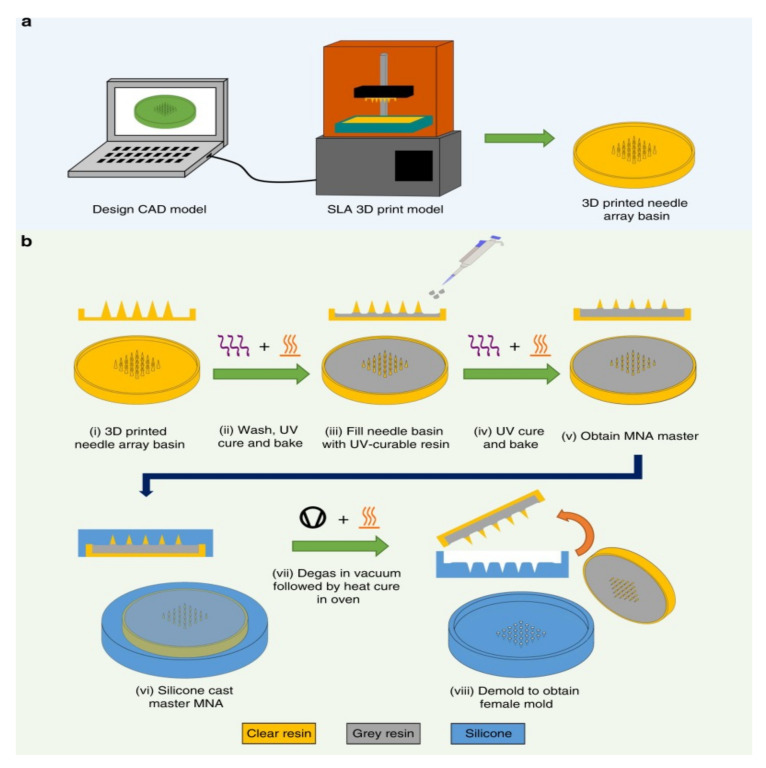
Overview of “Print and Fill” fabrication method: (**a**) Needle array basin design followed by 3D printing of the design using a Form 2 SLA printer. (**b**) MNA master mold fabrication method (i) take 3D printed needle array basin; (ii) washing followed by UV curing and baking of printed needle array basin; (iii) filing of needle array basin with UV-curable resin; (iv) second UV curing and baking; (v) obtain MNA master; (vi) silicone casting of MNA master; (vii) silicone mold is degassed followed by heat cure in oven; (viii) demolding to obtain usable MN mold [63]. Reproduced with permission from Kevin J. Krieger et al., Simple and customizable method for fabrication of high-aspect ratio MN molds using low-cost 3D printing; published by Springer Nature, 2019.

In recent years, additive manufacturing (3D printing) has rapidly been gaining attention as a means of producing MN arrays. A 3D printer builds an object by depositing the desired material layer-by-layer. The biomedical device industry has seen a rapid rise of 3D printing technologies in tissue engineering implants in recent years [153,154,155,156,157,158,159,160,161,162,163,164]. In 2019, Johnson et al. fabricated the first MN master using a commercial 3D printer [60]. Krieger et al. introduced a two-step called “print and fill” method to fabricate a MN mold (Figure 15) [63]. Another study used a stereolithography technique to fabricate MN patches [165]. An advantage of using 3D printers to manufacture a MN array is the flexibility of design parameters and compressed lead times for processing [60].

## 6. MN Mechanical Characterizations

During the MN design phase, it is critical to consider the mechanical properties of the MNs as they are subjected to an applied force for epidural insertion. To accomplish this, the MNs need to possess requisite strength in order to avoid failure in the MN array [166]. Lutton et al. argued that there is no single test that can simulate and observe the mechanical property of the needle and the insertion of the MN in vivo [167]. Consequently, a range of mechanical tests should be applied to the MN for characterization. Various types of mechanical tests on MNs include axial force, transverse force, base plate break, and insertion force (Table 4). Moreover, several investigations have been performed to study the relationship between mechanical characterization and MN manufacturing parameters [168].

### 6.1. Axial Force

The axial force test is the most common, and it consists of applying force to the tips of the needle in a vertical manner and to the base of the MN array [169]. This mechanical test is important and serves to determine the failure force of the needles. Knowing the failure force measurement of the needles is the best valuable information or called the safety point since it gives an approximate range (expectation) of needle insertion force [149].

**Figure 16:**
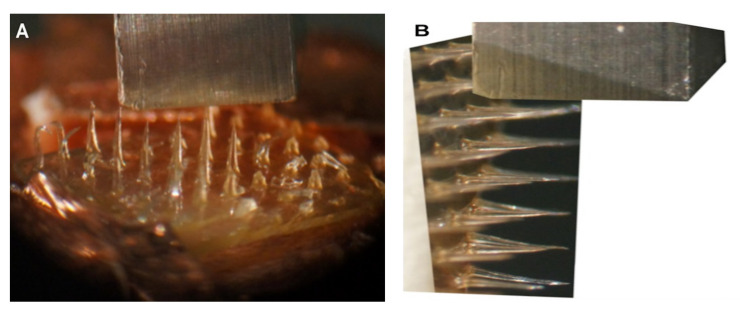
(**A**) Digital photograph of SA MN pressed against the metal mill during axial fracture force measurement with the micromechanical tester (Instron^®^ Model 5969; Instron, Norwood, MA). (**B**) MN shafts were transversely pressed against the metal mill for measurement of the transverse fracture force by way of the micromechanical tester (Instron^®^ Model 5969, Instron, Norwood, MA) [80]. Reproduced with permission from Demir et al., Characterization of Polymeric MN Arrays for Transdermal Drug Delivery; published by PLoS One, 2013.

Several axial force studies have been performed to determine the failure force of MNs using different equipment and calculation methods. Davis et al. measured the failure (ScopeTest1, EnduraTEC, Minnetonka, MN, USA) by calculating the force and displacement data [170]. Moreover, Demir et al. measured the fracture force by using a universal testing machine (Instron^®^ Model 5969, Instron, Norwood, MA, USA) (Figure 16A) [80]. Moreover, Khanna et al. studied the axial fracture tests using a compression load cell (LCFA-500gF sensing capacity, Omega Co., Norwalk, CT, USA) and motorized actuators (Z600 series Thorlabs Motorized Actuators, Morganville, New Jersey, USA) [171]. Donnelly et al. applied a compression mechanical tests by using a TA-XT2 Texture Analyzer (Stable Microsystems, Haslemere, UK) with the help of a light microscope (GXMGE-5 digital microscope, Laboratory Analysis Ltd., Devon, UK) [172]. Park and Prausnitz measured the failure test using a displacement-force test station (Model 921A, Tricor System, Elgin, IL, USA) [173].

### 6.2. Transverse Force

The transverse force test involves the application of a force parallel to the MN base plate with the y-axis. The irregularity of the skin surface may lead to transverse bending of the MN, and thus the measurement of the transverse fracture force is important [167]. Furthermore, along with the axial force, the transverse force completes the big picture of the MN’s mechanical property and thus predicts MN bending behavior during insertion [80]. The limitation of this test is that the metal probe has to be manually aligned with a defined length of the MN [167].

Donnelly et al. measured the transverse failure force of MN arrays using TA.XT-plus Texture Analyser (Stable Micro Systems, Surrey, UK) [169]. Another study was performed by Park et al. to measure the transverse force using a force–displacement station and a microscope [174]. The MN was set vertically on a metal plate with perpendicular loading by a PDMS structure. The transverse force was tested until the MNs were broken therefore concluding that displacement increases linearly with a MN base diameter. Demir et al. tested the transverse force of the MN by using a micromechanical tester (Instron^®^ Model 5969; Instron, Norwood, MA, USA) [80] (Figure 16B).

### 6.3. Insertion Test

**Figure 17:**
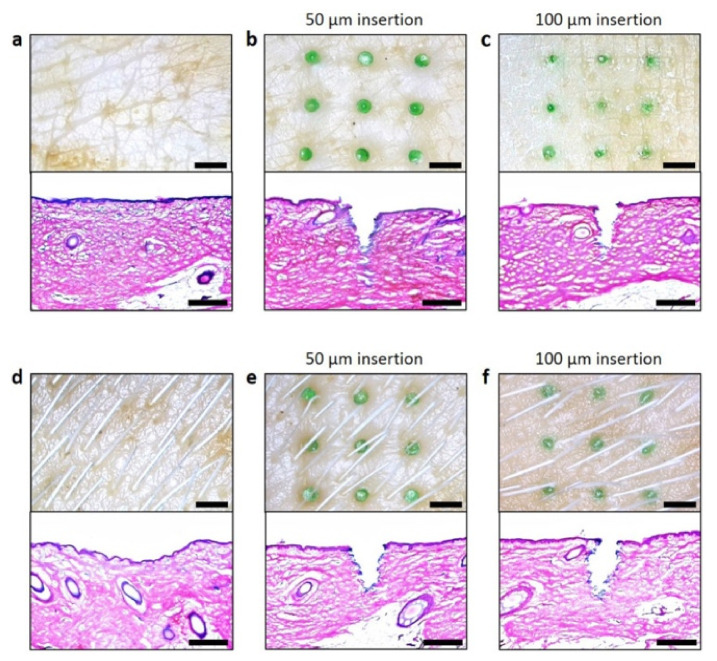
Microscopy images and histological examinations of hairy and hairless pig cadaver skin: (**a**) Hairless pig cadaver skin before DMN insertion. (**b**) 50 μm insertion of 600 μm tall DMN. The DMN was inserted 650 μm deep into the skin. (**c**) The base area of the DMN that was inserted 100 μm deep was less apparent on the skin surface compared with those inserted 50 μm deep. Histological examination showed that the DMNs were inserted 700 μm deep into the skin. (**d**) Hairy pig cadaver skin before DMN insertion. (**e**) The appearance of DMNs inserted 50 μm deep into hairy skin was similar to the appearance of DMNs inserted into hairless pig cadaver skin. (**f**) DMNs inserted 100 μm deep into the hairy pig cadaver skin penetrated 700 μm deep. Scale bars: microscopy images, 2 mm; histological images, 500 μm [175]. Reproduced with permission from Shayan F. Lahiji et al., A patchless dissolving MN delivery system enabling rapid and efficient transdermal drug delivery; published by Springer Nature, 2015.

**Figure 18:**
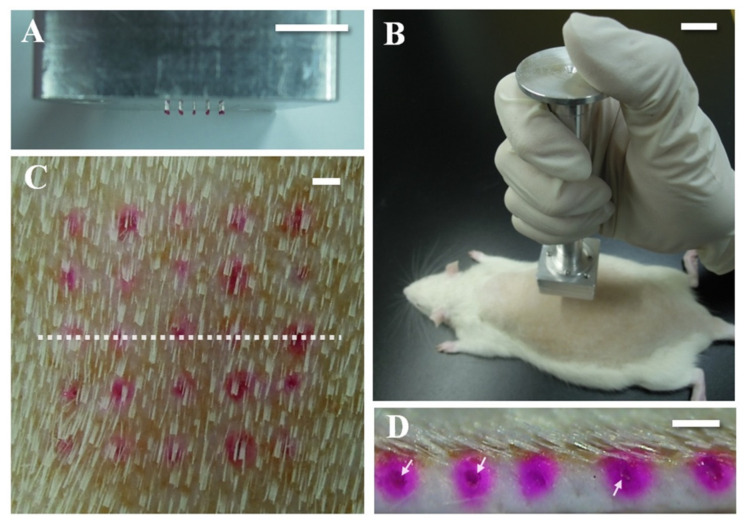
In vivo skin penetration study: (**A**) Troy MNs were assembled with an applicator into an array (5 × 5). (**B**) The applicator was applied to rat dorsal skin vertically by hand. (**C**) Image of skin with applied Troy MNs. The array of red spots indicates the penetrated site of rhodamine B-encapsulated Troy MNs and the white dotted line represents the vertically sliced line used to obtain sectional tissue. (**D**) Skin sectional image. Red spots mark delivered rhodamine B in the skin and the white arrow indicates undissolved parts of DMNs. Scale bars, 10 mm (**A**,**B**) and 1.0 mm (**C**,**D**) [176]. Reproduced with permission from Kim et al., The Troy MN: A Rapidly Separating, Dissolving MN Formed by Cyclic Contact and Drying on the Pillar (CCDP); published by PLoS One, 2013.

The insertion test is more important and different from the axial force as the axial force does not give an accurate measurement as the insertion test. Furthermore, different skin subjects were targeted in this test which include pigs (Figure 17), rats (Figure 18), and humans. One advantage of using a MN is the ability to load the drug and deliver it to the skin. Despite having several mechanical tests simulate the fracture force of the needle, it is important to validate the results with an actual skin.

Lee et al. applied a pyramidal MN into a full-thickness cadaver pig skin (series 8900, WHAL, Sterling, IL, USA) with a force of 1.5 N [177]. After that, a microscope (SZX12, Olympus, Bethlehem, PA, USA) was used to obtain a better look at the MN’s imprints. Donnelly et al. inserted a MN attached to a movable cylindrical probe into the skin of a stillborn piglet [172]. After that, the skin surface was observed with a digital microscope. Jun et al. measured the transverse compression load using a zwickiLine material testing machine (Z5.0TN, Zwick/Roell, Ulm, Germany) [178]. Further, Khan et al. applied different MN forces into a neonatal porcine skin to study the insertion depth using a texture analyzer [179]. Different insertion test studies were performed on three Caucasian male skins by using (Model 921A, Tricor Systems, Elgin, IL, USA) done by Davis et al. [170]. Another study applied optical coherence tomography (OCT) technology in scanning the depth of the MN in human skin [180].

**Table 4:** Overview: MN Mechanical Characterization.

	Description	Importance	Limitation	References
Axial Force	Apply force into the tip of the needle in vertical way (x-axis)	Determine the failure force of the tip needle.	Simulation (not accurate)	[80,157,176,177,178,179,180]
Transvers Force	Apply force into the MN base in parallel way (y-axis)	Determine the failure force of the needle base.	Simulation (not accurate)	[80,174,176,181]
Insertion Test	Apply the needles into a rat, pig, or human skin.	Determine the actual force on skin.Ability to release the drug.	Required a skin resource	[177,179,182,183,184,185,186,187]

## 7. MN Applications

MNs have attracted extensive interest by researchers, scientists, and industry participants. Several studies have demonstrated the potential and ability to administrate MN in different fields. These include drug delivery, vaccine delivery, disease diagnostic, and cosmetics application.

### 7.1. Drug Delivery

The first application of MN for drug delivery was by using a solid silicon MN in 1998 [57]. A dissolvable MN patch was used to deliver human growth hormone for transdermal delivery to hairless rat skin [181]. A dissolvable caffeine loaded MN patch was able to control the weight of obese mice and work as an anti-obesity treatment plan [182]. A coated MN patch was used to deliver salmon calcitonin [183]. A solid microneedle was used to deliver a protein antigen (ovalbumin) into hairless guinea pig skin [184]. Solid silicon and metal MNs were used for the delivery of calcein [185], BSA, and insulin [74]. Furthermore, MNs have been used for transdermal permeation for several drugs such as ibuprofen, ketoprofen, and paracetamol [186]. Other drugs administrated via microneedles include L-Ascorbic acid, riboflavin, aspirin, docetaxel, pilocarpine, lidocaine, hydrochloride, ketoprofen, and glycerol [187]. Despite the fact that most studies used microneedle array for drug delivery into mice, pig, human skin, there were other studies which successfully demonstrated microneedle injection into chicken thigh [188], and brain tissue [189].

### 7.2. Vaccine Delivery

A dissolvable MN is a common type of MN used for vaccine delivery purposes. The dissolvable MNs were used to replace hypodermic injection needles that were typically used to administer vaccines. Unlike other types of MN, the dissolvable MNs are biocompatible, robust, scalable, and do not generate biohazardous waste [190]. Dissolvable MNs were used to deliver vaccines for malaria, diphtheria [191], influenza [192], Hepatitis B [193], HIV [194], and polio [195].

Even though dissolvable MNs are most frequently used for vaccine delivery, coated MNs arrays have also been successfully used for vaccination purposes [53]. A study used a simple, safe, and compliant vaccination method to improve the immune system for pigs by administering bacillus Calmette–Guérin (BCG) vaccine with a coated MN [196]. Another study successfully encoded hepatitis C virus protein in DNA vaccine coated on microneedle [197]. The microneedle was effectively primed for specific cytotoxic T lymphocytes (CTLs) in mice. Furthermore, a coated microneedle carried influenza virus antigen for vaccination application in mice [198].

Hollow MNs have been used to deliver anthrax recombinant protective antigen vaccine to a rabbit instead of regular injection [199]. A hollow microneedle was evaluated for vaccination against plaque in a mouse model [200]. A clinical trial conducted in humans using hollow microneedle with influenza vaccination showed similar results with the immune system when compared to intramuscular injection [201].

### 7.3. Disease Diagnosis

Disease diagnosis and therapeutic efficacy can be monitored via several established bioassays that sample body fluids to assess and monitor health conditions. The current methods induce pain, require specialized techniques, tailored equipment, and professional medical personnel [202]. However, microneedle technology offers bioassays solution with painless experience and simple implementation [202].

A hollow MN has the ability to diagnose several diseases such as cancer [192], diabetes [203], and Alzheimer’s [204] disease. Patient health monitoring is another application of the MNs. For example, a hollow glass MN may be used to investigate the glucose level [205]. Furthermore, O’Mahony et al. proposed the MNs system for electrocardiography signal optimization [206]. A microneedle-based enzyme was functionalized to monitor alcohol in artificial interstitial fluid [207]. Microneedles with nanoparticles were able to identify the biomarkers in early stage of osteoarthritis [208]. Microneedles were used as sensors for hydrogen peroxide, lactate, dissolved oxygen, and glutamate [187].

### 7.4. Cosmetic Application

**Figure 19:**
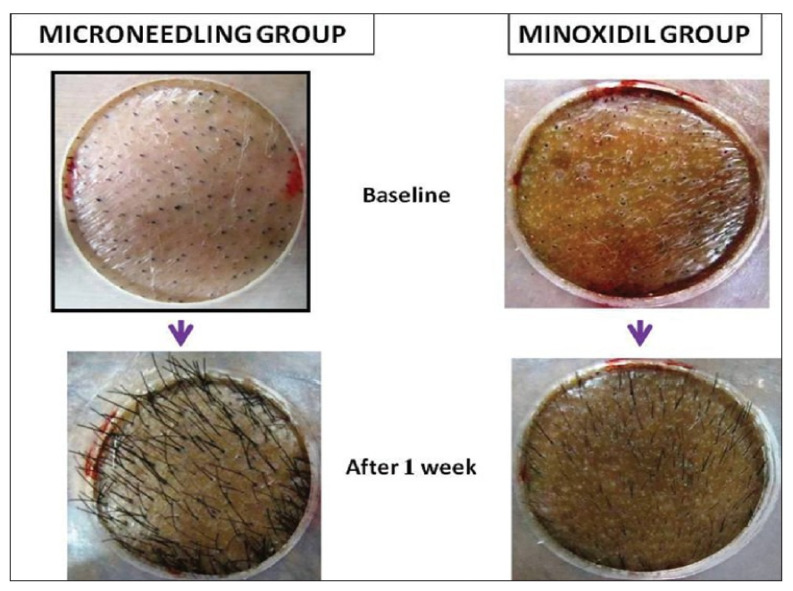
Faster hair re-growth at 1 week noted in Microneedling treated group [215]. Reproduced with permission from Rachita Dhurat et al., A Randomized Evaluator Blinded Study of Effect of Microneedling in Androgenetic Alopecia: A Pilot Study; published by International Journal of Trichology, 2013.

MNs have widely been used in cosmetic applications such as skin treatment and hair growth (Figure 19). Kim et al. developed a hyaluronic acid-based dissolvable MN patch for the intradermal delivery of ascorbic acid and retinyl retinoate [209]. Kumar et al. showed an enhancement of local delivery of eflornithine (used to reduce facial hirsutism) in vitro and in vivo using a solid MN [210]. Further, MN technology was able to treat two patients suffering from alopecia areata disease [211]. These patients experienced hair growth after treatment. Effective clinical trials have been conducted in atrophic facial scarring [212], atrophic acne scars [213], and hypertrophic burn scars [214] using a MN. Microneedles are considered as an effective treatment for cosmetic applications related to aging, skin lesions, vulgaris, and wrinkles [208]. With an increasing demand of cosmetic products, microneedles (patches and rollers) have a high potential in the future [194].

## 8. Gaps in Research and Future Outlook

This paper illustrates the benefits of MN utilization for several applications in comparison to other techniques. Moreover, several studies suggest different manufacturing methods, materials, and needle types for the fabrication of a MN array. A massive clinical trial was proposed for the adoption of MNs for different usage purposes. However, there are still gaps in the field of MN array production. In this section, we present a future outlook for MNs with respect to scale-up of manufacturing processes, predictive modeling of materials and manufacturing methods, and next-generation methods including additive manufacturing and usage towards the COVID-19 testing and vaccination.

### 8.1. Manufacturing Process Scale-Up

With the recent increase in the application for MNs and shortage of commercial MN products (only 13 MNs products are currently available) [216], the potential for large-scale MN manufacturing is in high demand. Bhatnagar et al. have stated that possessing more knowledge about manufacturing materials and chemistry will help industries achieve their financial goals, along with large-scale production [217] and increased profits. Most current MN fabrication methods require a number of steps to produce a single MN array [53]. Tackling this limitation unlocks future research opportunity regarding the reduction or integration of the number of processes required to fabricate a MN.

### 8.2. Predictive Model for MN Manufacturing

The design parameters for the fabrication of MNs lacks detailed understanding and warrants further exploration. Chung and Tu extended their study on fabricating the MN array to integrating CO_2_ laser processing and polymer molding by studying the PDMS MN’s dimensions with different laser power and scanning speed values [218]. Aoyagi et al. studied the influence of pulse shot number and hole diameter on fabrication depth. Furthermore, they noted an influence of repetition rate and hole diameter on sidewall smoothness [126]. However, there was no study investigating fabrication approaches to MNs arrays based on the manipulation of process parameters. Several types of predictive models have been used in literature to improve the performance of manufacturing processes [219,220,221,222,223,224] which can be extended for MN fabrication. Prior literature has demonstrated that computational modeling [225,226,227,228,229,230] coupled with novel manufacturing processes can deliver complex biomolecules for biomedical applications. Furthermore, a predictive model is required that can relate fabrication parameters to drug elution properties as well as sensing applications.

### 8.3. Next Generation of MN

Different studies have conducted in vivo studies of a fabricated MN to deliver drugs and vaccines. However, an upcoming challenge is the fabrication of a MN that has the ability to deliver macromolecules with high molecular weights and high hydrophilicity [69,231,232,233]. Other issues associated with using a MN for drug delivery are irritation, skin allergy, and redness [16]. Different MN devices such as Dermaroller are available in the market, however no biodegradable polymer MN is being commercialized yet [234]. Moreover, commercially there exist no MN that can incorporate protein products [235].

### 8.4. MN and Additive Manufacturing

Additive manufacturing is a promising technology that offers a high-quality resolution, low cost, and less fabrication time [236,237]. Using a 3D printer to fabricate a MN array would be advantageous given that device dimensions and formulation can be modified with minimal postprocessing steps when compared to conventional MN manufacturing methods. In addition, direct-write processes [238,239,240,241,242,243,244,245,246] can be utilized to coat the MNs with different biomolecules for efficient drug release. Recently, the first study that used a commercial 3D printer to manufacture a MN structure was done by Johnson and Procopio [60]. They used an Autodesk Ember printer with optimized antialiasing and lower layer height to produce fine needles. Another recent study was done using an affordable SLA 3D printer to fabricate MN array [63]. This study was extended to test the insertion of MN into the skin by applying a force of 30N.

### 8.5. Covid19 and MN

With the impact of the Coronavirus (Covid19) pandemic being felt worldwide, the MN approach serves as a good candidate in fighting the pandemic. Chen et al. introduced a MN-based oropharyngeal swabs to be used to reduce the false negatives in COVID-19 testing [247]. This concept allows the doctors to identify between positive and negative samples by capturing the virus with high efficiency. As the COVID-19 vaccine is available these days, the vaccine could be carried in MNs and distributed to people who are able to self-administer the vaccine.

## 9. Conclusions

The importance of overcoming the stratum corneum barrier is central to efficient MN-mediate transdermal and intradermal delivery. This paper summarizes MNs technology in the transdermal drug delivery era. Extensive studies and research have been conducted in the fabrication of MNs due to its advantages. Various MN design types, material, and manufacturing methods have been illustrated in this paper.

Over the past few decades, a variety of MN systems with distinct delivery mechanisms have been developed and used for the delivery of small or macromolecules. Recent investigations showed that temporary disruption of the skin microchannel lifetime enhances transdermal delivery efficiency of small molecular drugs, salt forms, excipients, and other formulation factors as highlighted in this comprehensive review. Intradermal and transdermal delivery of macromolecules including therapeutic peptides and proteins, vaccines, and synergistic effect of combined enhancement in addition to MN treatment were briefed. Moreover, MN mechanical tests and their characterization are explored in the literature.

Finally, this paper exposes the gap in research for MN fabrication. Although there are several new transdermal products mediated by MNs, these, however, have not reached full maturity. As the understanding of MN-mediated advances, it becomes increasingly evident that there is a gap in enabling cost-effective manufacturing for large-volume production of MNs.

## Figures and Tables

**Table 3:** Overview: MN Manufacturing Method.

Manufacturing Method	Description	Advantages	Disadvantages	References
Laser Ablation	Uses a focused optical light beam to fabricate a MN array on a substrate.	Less time consuming.	Might cause a crack or fatigue resistance on the substrate (MN array). High cost. Not suitable for large production.	[109,118,120,121,122,123,124,125,126,127,128,129,130,131,132,133,134,135,136,137,138,139,140]
Lithography	Transfers the master pattern of the geometric shapes onto the surface of a substrate.	Produces MN from a variety of material. Very precise geometries Smooth vertical sidewall.	Time consuming.	[53,118,134,141,142,143,144,145,146,147,148,149,150,151,152,153]
Micro-molding	Replicates a master mold and casts the mold with a solution.	Used for mass production. Cost effective.	Controls the depth of penetration. Drug load capacity. Mechanical behavior.	[14,154,155,156,157]
Injection molding	Injecting molten plastic materials into a mold.	Mass production.	High initial cost (machine equipment cost). Complex processes.	[67,80,107,158,159]
Additive manufacturing	Printing the MNs layer by layer.	Customizable Design.	Requires a high-quality 3D printer. Offer limited accuracy.	[60,63,160,161,162,163,164,165,166,167,168,169,170,171,172]

## Data Availability

The data presented in this study are available on request from corresponding author.
